# Supplementary material for: Traffic-Related High Sleep Disturbance in the LIFE-Adult Cohort Study: A Comparison to the WHO Exposure-Response-Curves
Source: Int J Environ Res Public Health. 2023 Mar 10;20(6):4903. doi: 10.3390/ijerph20064903 (PMC10049209; doi:10.3390/ijerph20064903)
Supplement: Supplementary file 1 [file ijerph-20-04903-s001.zip › ijerph-2189569-supplementary.pdf]

Table S1. Description of study population with regard to HSD from road, rail and air traffic.

		Road				Rail				Air			
		Not HSD		HSD		Not HSD		HSD		Not HSD		HSD	
		n	%	n	%	n	%	n	%	n	%	n	%
Total		5002	97.3	138	2.7	4908	98.6	71	1.4	4931	98.0	101	2.0
Sex													
	Females	2623	96.9	85	3.1	2343	98.9	27	1.1	2348	97.7	56	2.3
	Males	2379	97.8	53	2.2	2565	98.3	44	1.7	2583	98.3	45	1.7
Age (years) at baseline													
	18-24	0	0	0	0	0	0	0	0	0	0	0	0
	25-29	35	100	0	0	33	97.1	1	<0.1	34	100	0	0
	30-34	65	98.5	1	1.5	66	98.5	1	1.5	66	98.5	1	1.5
	35-39	65	95.6	3	4.4	67	100	0	0	67	98.5	1	1.5
	40-44	76	95.0	4	5.0	76	96.2	3	3.8	79	98.8	1	1.3
	45-49	445	97.2	13	2.8	441	98.7	6	1.3	448	98.7	6	1.3
	50-54	678	96.4	25	3.6	685	98.8	8	1.2	676	97.0	21	3.0
	55-59	611	97.1	18	2.9	609	98.7	8	1.3	606	97.0	19	3.0
	60-64	540	97.5	14	2.5	530	98.1	10	1.9	536	98.2	10	1.8
	65-69	697	98.2	13	1.8	673	97.8	15	2.2	682	98.3	12	1.7
	70-74	647	96.9	21	3.1	632	98.8	8	1.3	635	98.3	11	1.7
	75-80	720	97.6	18	2.4	695	99.0	7	1.0	703	98.5	11	1.5
	80+	423	98.1	8	1.9	401	99	4	1.0	399	98.0	8	2.0
Socioeconomic class													
	Low	763	96.3	29	3.7	753	98.0	15	2.0	753	98.0	15	2.0
	Moderate	3053	97.3	86	2.7	2980	98.5	45	1.5	3002	97.9	64	2.1
	High	1179	98.1	23	1.9	1168	99.1	11	0.9	1169	98.2	22	1.8
	Missings	*7*		*0*		*7*		*0*		*7*		*0*	

Table S2. Example for % highly sleep disturbed (HSD) from railway traffic noise for the LIFE-Adult study and the WHO Review by Basner & McGuire 2018 [14].

L_{night}	%HSD from rail traffic noise	
	WHO	LIFE-Adult
35 dB	-[1]	1
45 dB	3	1
55 dB	11	4
65 dB	26	9

[1] The WHO models had a noise level limit of 40 dB

Table S3. Example for % highly sleep disturbed (HSD) from traffic noise for the LIFE-Adult study (using second-order polynomial models derived from logistic regression ORs) and the WHO Review by Basner & McGuire 2018 [14].

L_{den}	Road traffic noise			Railway traffic noise			Aircraft traffic noise		
	WHO Review	LIFE-Adult (prevalence)*	LIFE-Adult (odds)**	WHO Review	LIFE-Adult (prevalence)*	LIFE-Adult (odds)**	WHO Review	LIFE-Adult (prevalence)*	LIFE-Adult (odds)**
**35 dB**	-	1	1		2	3		2	5
**45 dB**	3	2	1	3	1	0	15	32	32
**55 dB**	6	5	4	11	4	4	26	36	47
**65 dB**	12	10	11	26	10	15	40	-[1]	-[1]

*second-order polynomial equations obtained directly from original values
**second-order polynomial equations obtained from predicted values coming from logistic regression, same as the methodology used in the WHO review
[1] None of the participants in the LIFE Adult study were exposed to aircraft noise levels of 65 dB and more.

Figure S1: Official noise map of the city of Leipzig for 2012 (https://static.leipzig.de/fileadmin/mediendatenbank/leipzig-de/Stadt/02.3_Dez3_Umwelt_Ordnung_Sport/36_Amt_fuer_Umweltschutz/Luft_und_Laerm/Laermschutz/Laermkartierung/Immissonskarten/Laerm_Gesamt_LDEN.pdf).

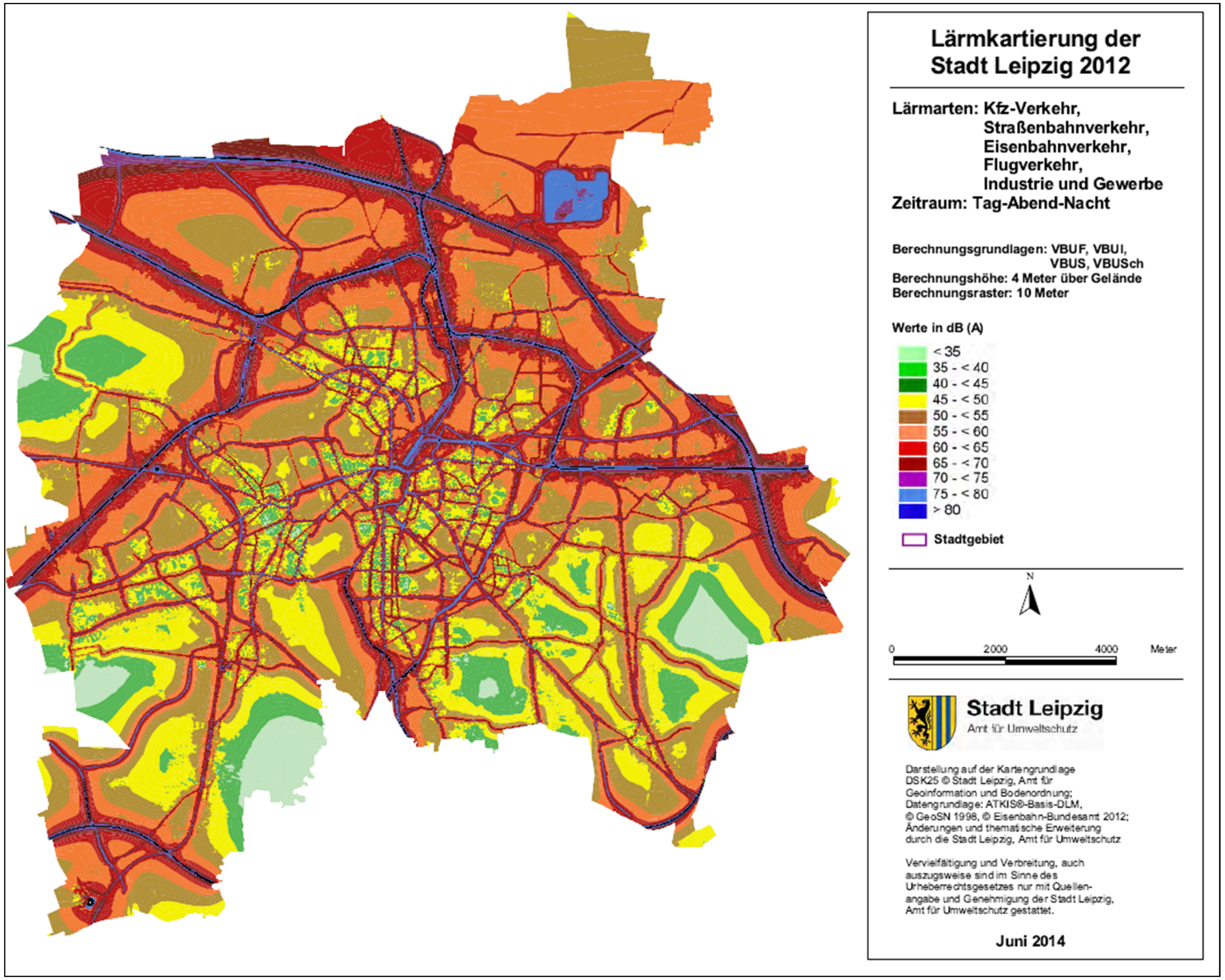

Figure S2: Comparison of HSD risk curves for rail traffic noise between the LIFE study and the WHO (Basner & McGuire 2018 [14]).

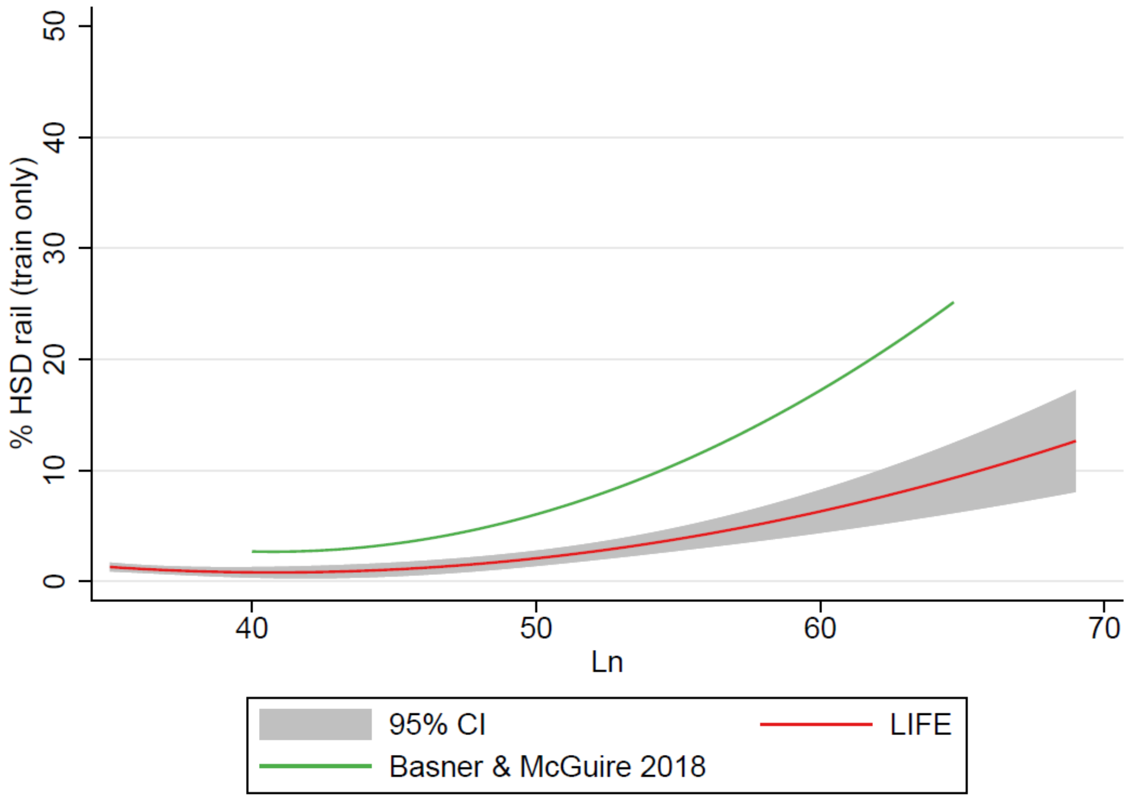

Figure S3: Comparison of % highly sleep disturbed (HSD) for traffic noise between the LIFE-Adult study and the WHO review by Basner & McGuire 2018 [14], using predictions from the univariate logistic regression model [52].

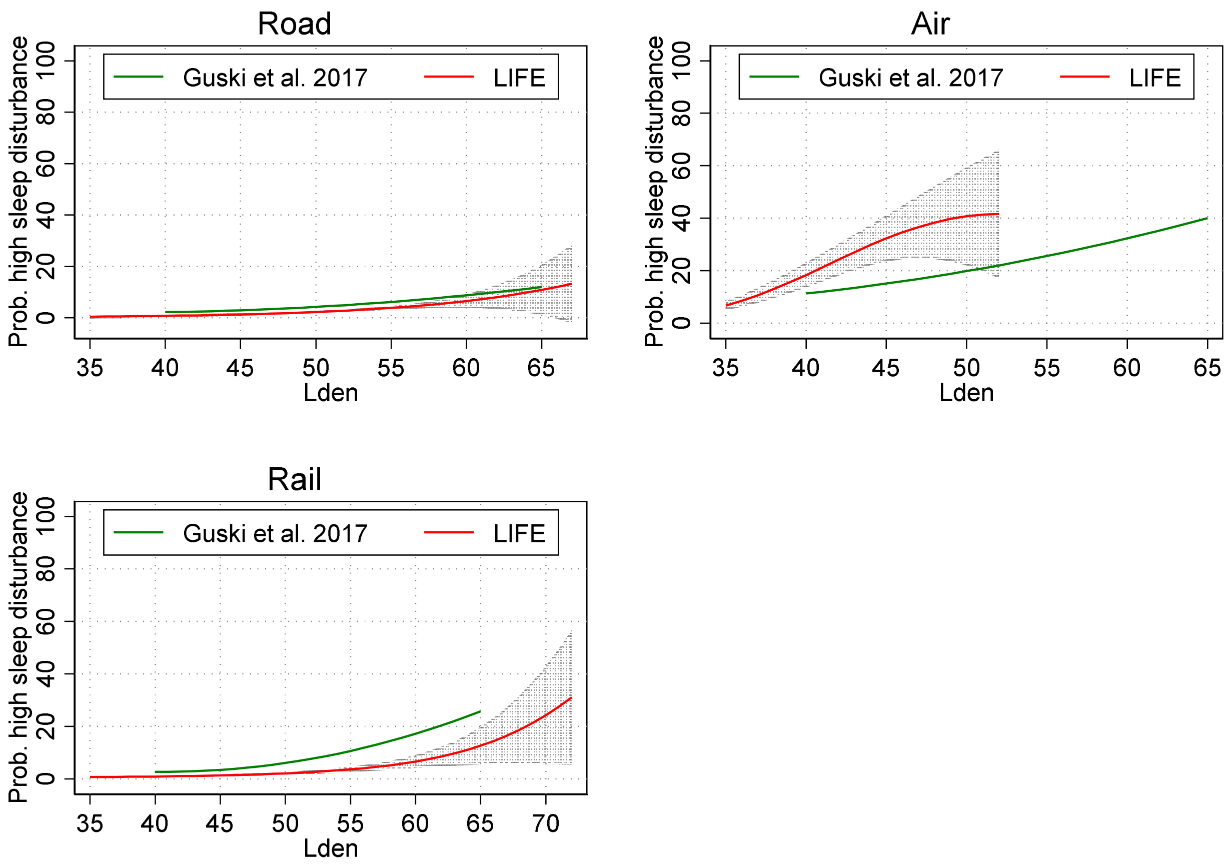

Road%HSD = 27.6428 – 1.3393* L_{night} + 0.0166* L_{night}^2
Rail%HSD = 72.4615 – 3.2453*L_{night} + 0.0362*L_{night}^2
Air%HSD = --181,7182+ 7.3837*L_{night} – 0.0588*L_{night}^2
